# Confocal microscopy reveals nerve fiber similarities in fibromyalgia and patients with dry eyes with a normal ophthalmic exam

**DOI:** 10.1186/1744-8069-10-S1-P2

**Published:** 2014-12-15

**Authors:** Daniel E Harper, Vincent Pallazola, David A Williams, Steven Harte, Daniel J Clauw, Roni M Shtein

**Affiliations:** 1 Department of Anesthesiology, University of Michigan, Ann Arbor, MI 48106, USA; 2 Department of Ophthalmology and Visual Sciences, University of Michigan, Ann Arbor, MI 48106, USA

## Introduction

Small fiber neuropathy has been shown in a subset of fibromyalgia patients, but this is a non-specific finding that has been noted in several chronic pain states. Small nerve fiber morphology can be measured non-invasively in the cornea, an area densely innervated with small fibers, using in-vivo confocal microscopy (IVCM). We used this technique to determine if 1) neuropathy is present in the corneas of fibromyalgia patients and 2) if those who suffer from idiopathic, chronic dry eyes (i.e., dry eye symptoms without dry eyes on exam) might also have compromised small fibers.

## Methods

Fibromyalgia patients (n=19), chronic dry eye sufferers (n=12), and healthy controls (n=24) underwent IVCM to measure the morphology of corneal nerve fibers. In addition, participants also received an ophthalmic evaluation to measure tear function.

## Results

ANOVA revealed significant differences across the three groups in corneal nerve length. Post-hoc tests showed that nerves were significantly shorter in fibromyalgia patients (mean = 1.85 mm), compared with both dry eye subjects (2.66 mm) and healthy controls (2.68 mm). When the dry eye group was broken down into subgroups of those displaying normal vs. decreased tear function, it was revealed that corneal nerves were significantly shorter in the dry eye subjects with a normal tear function (2.03 mm), compared to those with altered tear function (3.12 mm). Nerve length was not significantly different between fibromyalgia patients and dry eye subjects with normal tear function.

## Conclusions

Nerve length is significantly reduced in the cornea of fibromyalgia patients, indicating that small fiber abnormalities in fibromyalgia are not limited to the skin. More research is needed to determine whether these abnormalities are a cause or a symptom of the syndrome. Individuals who experience idiopathic dry eyes but whose tear function is normal also show evidence of corneal neuropathy, similar to fibromyalgia patients. These data suggest that idiopathic dry eyes may be an ophthalmic manifestation of centralized conditions such as fibromyalgia and other associated chronic overlapping conditions.

